# Use of the waxworm *Galleria mellonella* larvae as an infection model to study *Acinetobacter baumannii*

**DOI:** 10.1371/journal.pone.0283960

**Published:** 2023-04-05

**Authors:** Kah Ern Ten, Nazmul Hasan Muzahid, Sadequr Rahman, Hock Siew Tan

**Affiliations:** 1 School of Science, Monash University Malaysia, Bandar Sunway, Selangor Darul Ehsan, Malaysia; 2 Tropical Medicine and Biology Multidisciplinary Platform, Bandar Sunway, Selangor Darul Ehsan, Malaysia; CUHK: The Chinese University of Hong Kong, HONG KONG

## Abstract

*Galleria mellonella* larvae have been increasingly used in research, including microbial infection studies. They act as suitable preliminary infection models to study host-pathogen interactions due to their advantages, such as the ability to survive at 37°C mimicking human body temperature, their immune system shares similarities with mammalian immune systems, and their short life cycle allowing large-scale studies. Here, we present a protocol for simple rearing and maintenance of *G*. *mellonella* without requiring special instruments and specialized training. This allows the continuous supply of healthy *G*. *mellonella* for research purposes. Besides, this protocol also provides detailed procedures on the (i) *G*. *mellonella* infection assays (killing assay and bacterial burden assay) for virulence studies and (ii) bacterial cell harvesting from infected larvae and RNA extraction for bacterial gene expression studies during infection. Our protocol could not only be used in the studies of *A*. *baumannii* virulence but can also be modified according to different bacterial strains.

## Introduction

*Acinetobacter baumannii* has gained prominence as a leading healthcare-associated pathogen during the past few decades [1]. Increased morbidity and death due to infections by this microorganism place a significant expense on patients and healthcare facilities. According to the World Health Organization (WHO), multi-drug resistant (MDR) *A*. *baumannii* is known as a top-priority critical pathogen [2]. There are few virulence studies found for this bacterium. This may be because there is a lack of standardised disease models for determining the virulence of this important pathogen.

It is important to do research using animal models to gain a deeper understanding of human infections and the interactions between hosts and pathogens. Approximately 75 to 100 million vertebrates are currently used for scientific experimental reasons each year, and mice and rats are predominantly used [3]. There has been a growing public outcry in recent years over the excessive experimentation of animals [4]. This has led to stricter rules being implemented, including lengthy delays in approval because of the need for authorisation under ethical regulations. Working with vertebrate models requires specialised training, appropriate permissions and equipment, animal adaption times, and substantial expenditures, all of which complicate scientific study [5]. This led researchers to look for alternative animal models. Among mammalian models to study *A*. *baumannii* pathogenesis, murine models are still predominantly used [6]. However, a variety of other mammalian species, including rabbits, guinea pigs, and porcine models, have also been subjected to investigation [7].

It has been demonstrated that the use of insect models can assist in the comprehension of the pathogenicity of bacterial infections in human beings. Different invertebrate models are used to investigate host-pathogen interactions of *A*. *baumannii*. Some examples of these models are the larger wax moth *Galleria mellonella*, the worm *Caenorhabditis elegans*, the fruit fly *Drosophila melanogaster* and zebrafish (*Danio rerio*). Among them, *G*. *mellonella* has garnered an increasing amount of interest during the past decade [7]. *G*. *mellonella* larva has become a valuable insect model because it can survive at 37°C which mimics human body temperature, a condition necessary for demonstrating most human pathogens’ virulence factors. Besides, the larvae may be obtained at a low cost and are simple to keep alive using common household items. In contrast to the mammalian model, the *G*. *mellonella* model does not need ethical approval, and its rapid reproduction rate makes it ideal for high-throughput study. Although *G*. *mellonella* lacks an adaptive immune response, they have a rather sophisticated cellular and innate humoral immunity [8]. There are a lot of striking resemblances between this innate system in insects and mammals [9]. In the larvae, the cellular immune response comprises haemocytes, which produce an antimicrobial response by participating in phagocytosis, encapsulation, and clotting [10].

There are limited studies using the *A*. *baumannii*- *G*. *mellonella* infection system, and there are few established protocols for *G*. *mellonella* larval care. Therefore, we have developed a protocol for the use of *G*. *mellonella* in laboratories. This protocol can be used as a reference for scientists who are interested in studying the pathogenesis of *A*. *baumannii* using *G*. *mellonella* as a model. We have selected *A*. *baumannii* C98 (GenBank Accession: GCA_024357825.1), a virulent strain isolated from the Segamat community in Malaysia as a bacterial model.

Thus, the study aimed to establish a protocol for (i) rearing and maintenance of *G*. *mellonella* using simple housing equipment and food ingredients that can be purchased conveniently from local shops, (ii) infection assays to study *A*. *baumannii* virulence, and (iii) RNA isolation from larval hemolymph that can be used for downstream purposes such as transcriptomics studies.

## Materials and methods

The protocol described in this peer-reviewed article is published on protocols.io, dx.doi.org/10.17504/protocols.io.n92ldpr38l5b/v1 and is included for printing as a S1 File with this article.

### Expected results

**Fig 1:**
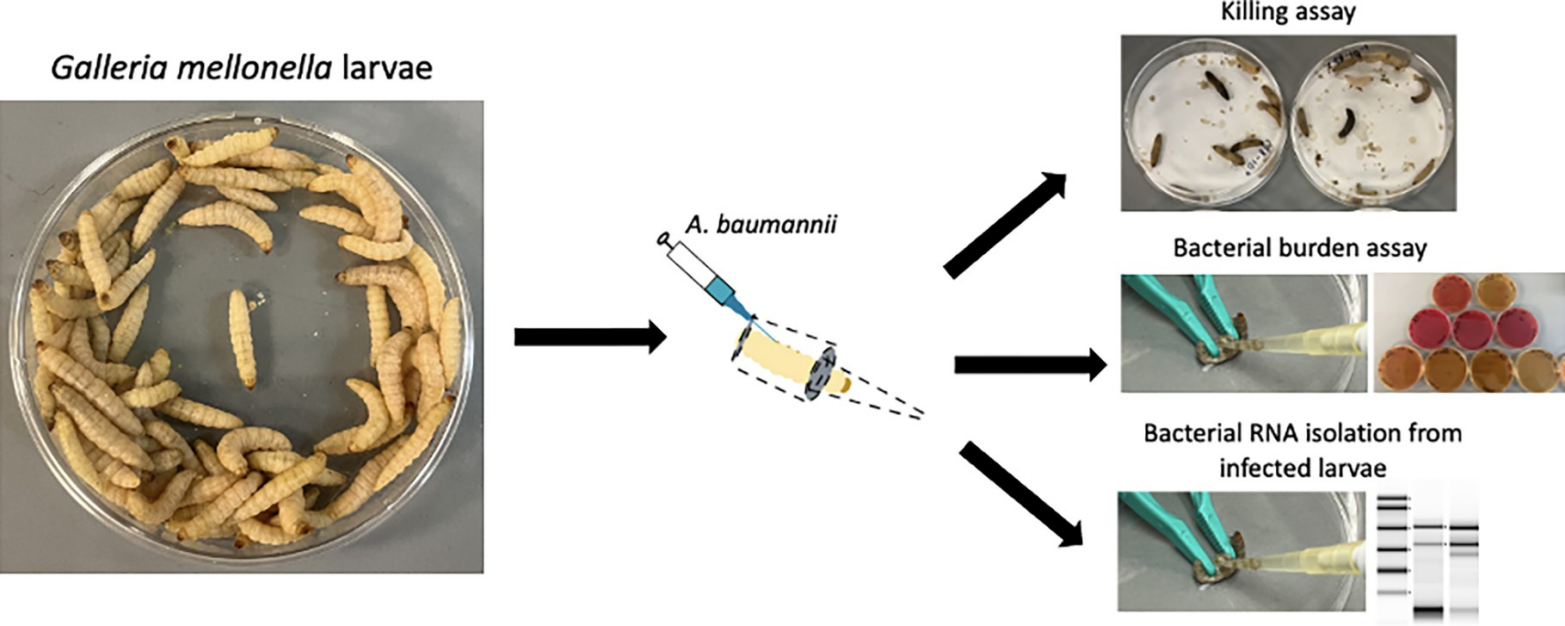
Summary of experiments included in this lab protocol.

**Fig 2:**
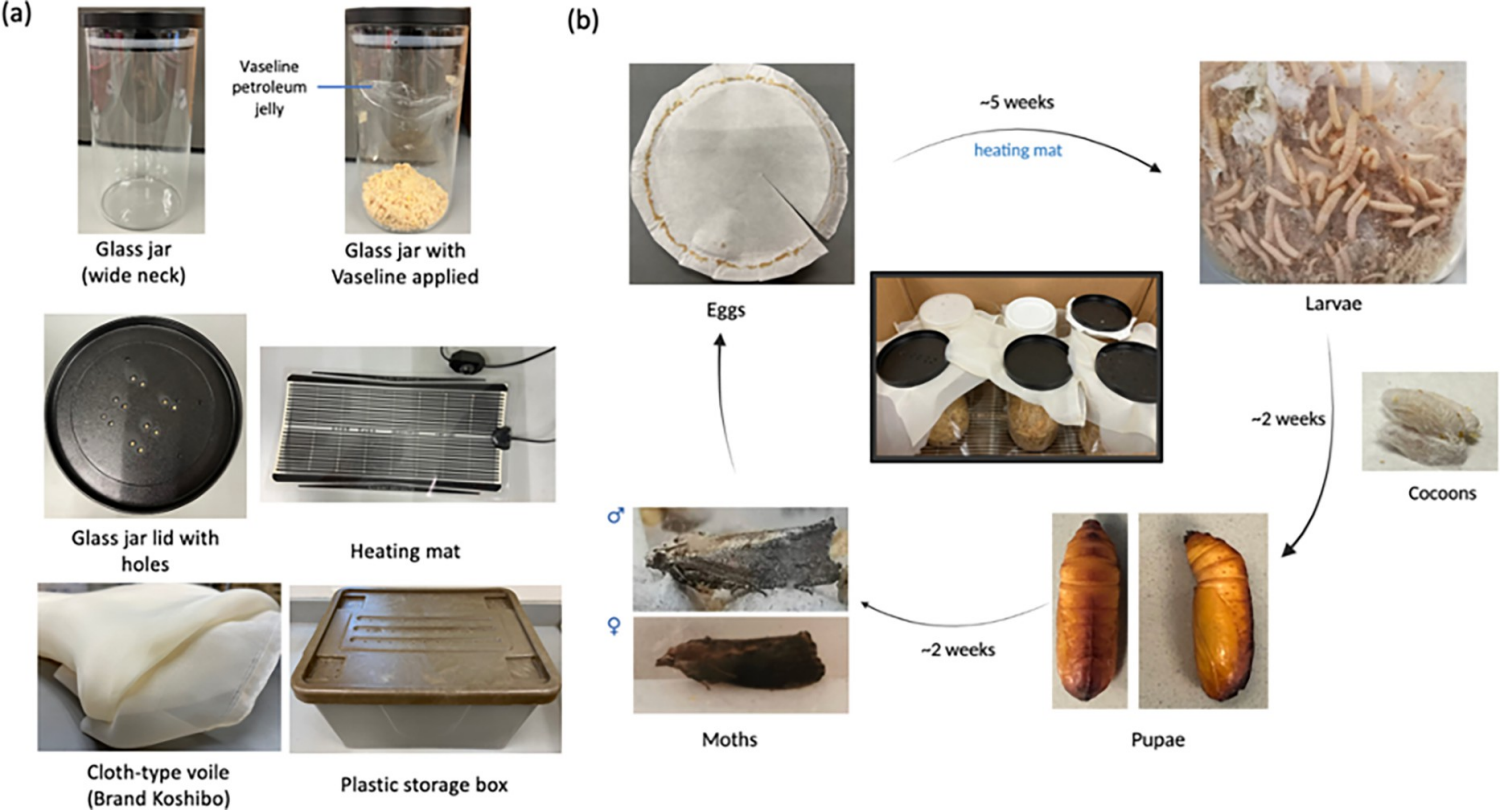
Rearing and maintenance of *G*. *mellonella* in the science research laboratory. (a) Housing equipment for *G*. *mellonella*. (b) The lifecycle of *G*. *mellonella* reared and maintained in our research laboratory. The larval stage of *G*. *mellonella* (eggs to last-instar stage larvae) was maintained at a temperature-controlled condition with a heating mat.

This lab protocol presents a simple and easy rearing and maintenance of *G*. *mellonella*, and bacterial infection studies using *G*. *mellonella* larvae as an infection model (Fig 1). They can be maintained in science research laboratories with simple and inexpensive equipment (Fig 2A). *G*. *mellonella* favours higher temperatures of 29–33°C [11], and the lifespan of *G*. *mellonella* can often be altered by adjusting the temperature of their living environment, where a shorter lifespan is observed at higher temperatures [12, 13]. However, maintaining *G*. *mellonella* at a temperature higher than room temperature may require special instrument, such as a temperature-controlled incubator, which might not be feasible for storing *G*. *mellonella* glass jars. This lab protocol described here utilises a cheap heat generator—a heating mat, which can be easily purchased from local shops. We compared the lifespan of *G*. *mellonella* by incubating them upon a heating mat with a controlled temperature at 32°C ± 2°C and humidity of 44%-54%, and at a room temperature of 22°C ± 2°C, with humidity 67%-72%. We observed that early-stage larvae (2^nd^ instar and 3^rd^ instar) appeared from freshly collected eggs after 3 weeks of incubation upon the heating mat. However, no hatched larvae were observed when incubating the eggs at room temperature. Therefore, using a heating mat allows an increased growth rate of *G*. *mellonella*. Generally, our *G*. *mellonella* took about 5 weeks from eggs to 6^th^ instar stage larvae suitable for experimental use (Fig 2B). It is recommended to apply a thin layer of Vaseline® petroleum jelly at the wall of the glass jar as newly hatched larvae are tiny and could easily escape from the holes of the lid, followed by a layer of filter paper covered with a perforated lid. The healthy, non-melanized larvae can be kept at room temperature without food in a dark environment, and they must be used for experiments within 2 weeks. Larvae in thick cocoons (pre-pupae stage) should be avoided for experimental use as they might cause inaccurate results.

**Fig 3:**
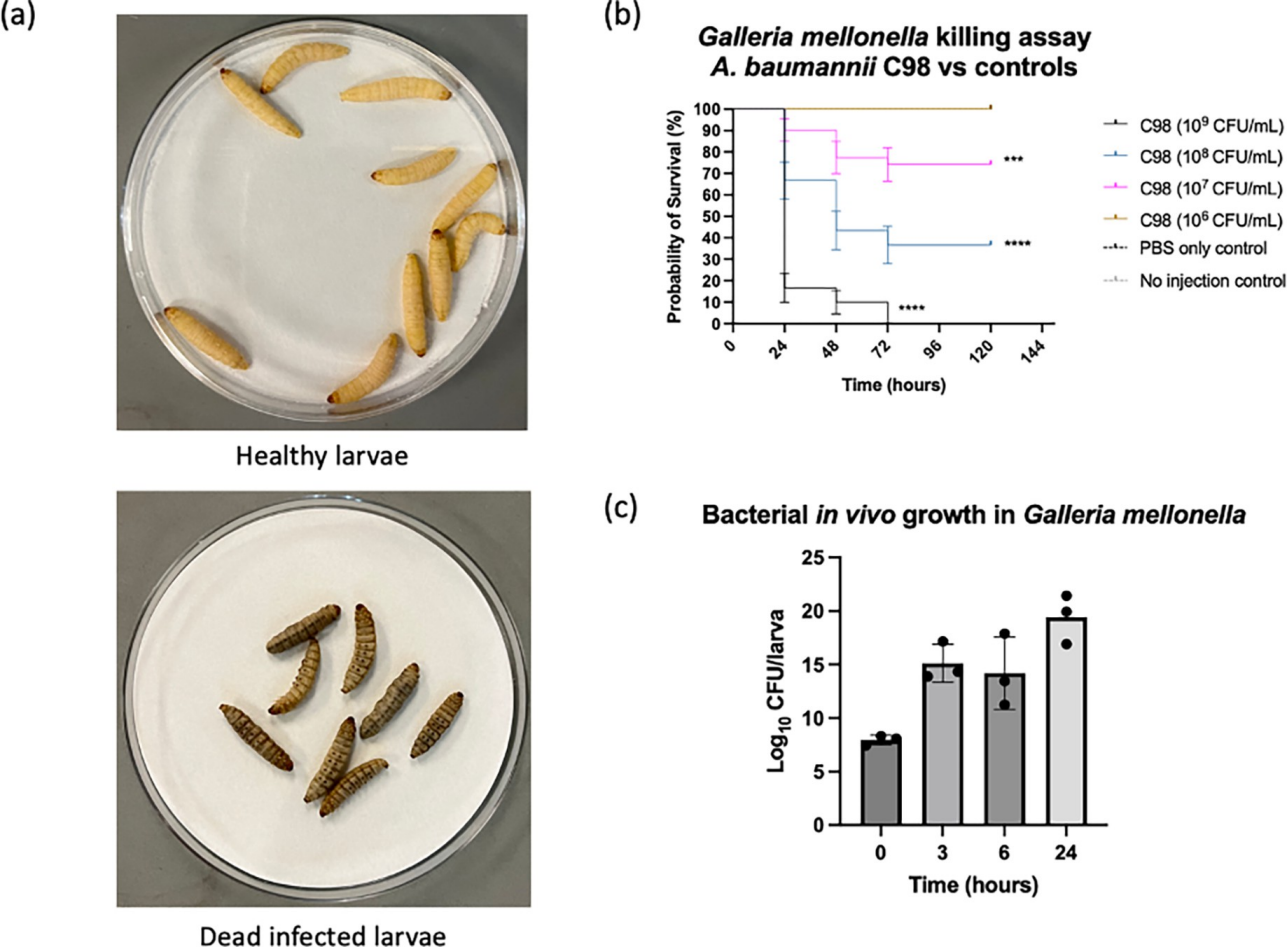
*G*. *mellonella* infection assays. (a) Healthy larvae have a white-creamy appearance with no melanisation. Dead infected larvae showed different levels of melanisation and were unresponsive to physical stimuli. (b) Kaplan-Meier survival curves of the killing assay. The results showed that *A*. *baumannii* strain C98 killed *G*. *mellonella* dose-dependently. (c) Bar chart of bacterial growth *in vivo*. The results demonstrated that *A*. *baumannii* strain C98 increased rapidly in the *G*. *mellonella* after 3 hours of incubation.

To study the *A*. *baumannii* virulence, here we describe an adapted protocol from Harding, Schroeder [14] with slight modifications. Prior to the infection assays, it is recommended to incubate the larvae at 37°C one day before the experiment as this allows the selection of more suitable larvae. In contrast, unhealthy larvae will show melanisation and/or death after the pre-incubation and will be excluded from the experiment [15]. Healthy and sick/infected larvae can be differentiated by their colours. Healthy larvae have a creamy-white appearance, while infected larvae will show different levels of melanisation (brown/grey/black colours) (Fig 3A). Prolonged incubation or starvation before the experiment should be avoided as this will alter the larval immunity [4]. To facilitate the injection process, a cut pipette tip can be used as a restraint device to trap the larva, and the larval prolegs can be exposed [16]. Fig 3B shows the results of the *G*. *mellonella* killing assay infected by serial dilutions of *A*. *baumannii* strain C98, where a bacterial concentration of 10^9^ CFU/mL caused 83.33% mortality after 24 hours of infection. The survival rates increased as the injected bacterial concentrations reduced. No larval death was observed in the control groups (PBS-only and no injection) throughout the experiments. We observed that pupation occurred after 5 days of incubation. Therefore, the experiments ceased afterwards to avoid biases.

Fig 3C demonstrates the results of quantifying the bacterial amount in the infected larvae. The hemolymph extraction was performed by puncturing the cuticle between the second and third proleg and collecting the hemolymph immediately via pipetting instead of cutting the larval tail. This allows as much of the hemolymph to be collected by gently squeezing the larvae using sterilised blunt-end plastic forceps and minimises the contamination from the larval gut microbiome. However, larval fat cells should be avoided when collecting the hemolymph. The bacterial CFU/larva can be calculated by the formula below:

$$CFU/larva=\frac{\frac{\left(\frac{number\;of\;colonies*dilution\;factor}{volume\;of\;diluted\;hemolymph\;used\;for\;plating}\right)}{volume\;of\;raw\;hemolymph\;used\;for\;serial\;dilution}*volume\;of\;raw\;hemolymph\;extracted\;(mL)}{number\;of\;larvae}$$

**Fig 4:**
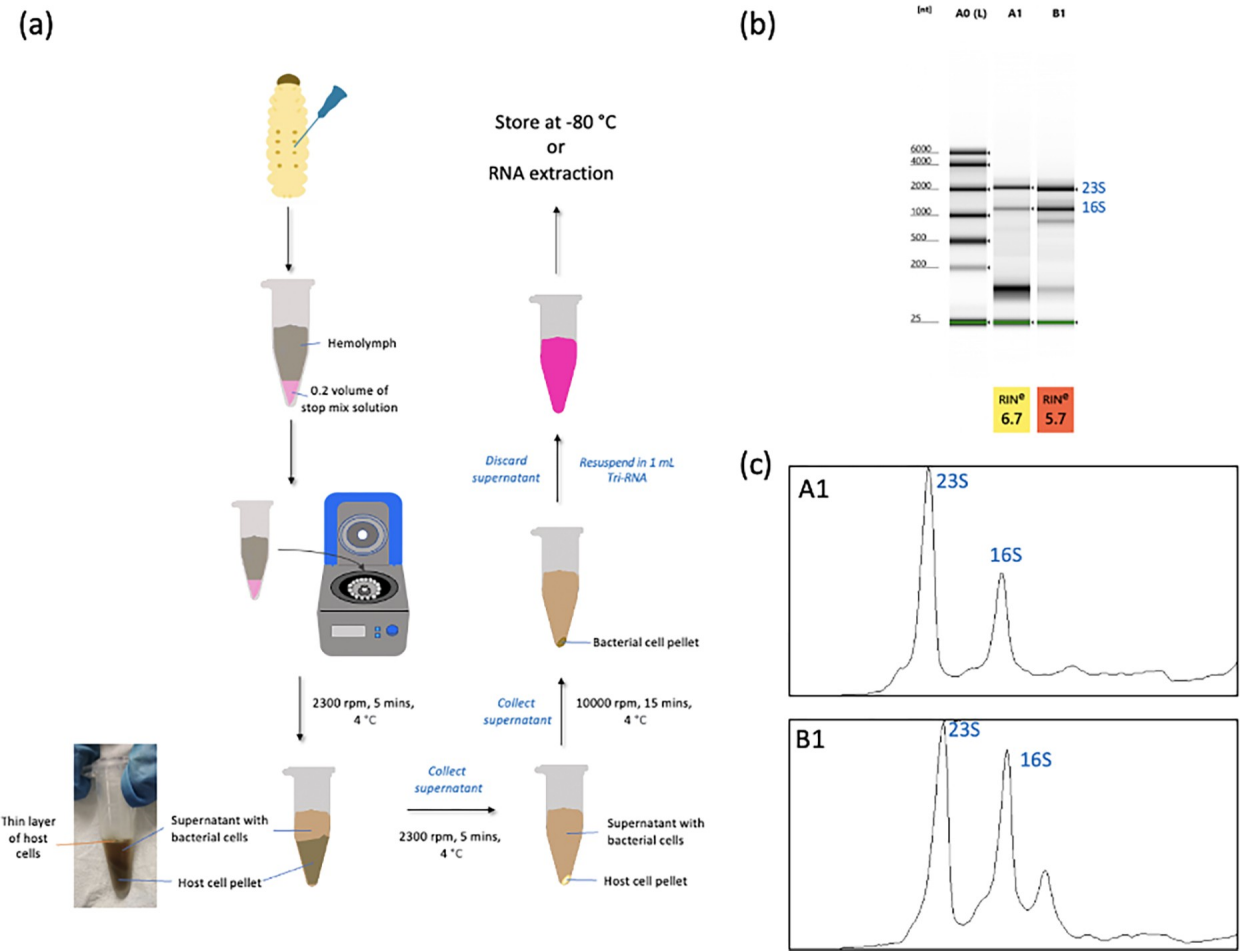
Bacterial RNA isolation from *G*. *mellonella* larvae infected by *A*. *baumannii* strain C98. (a) Steps of bacterial cell harvesting from the infected larvae. The host cells were removed by multiple centrifugations to minimize the contaminations of the host’s RNA. (b) Quality assessment of RNA samples using Agilent 2200 TapeStation System and the RIN numbers were calculated by TapeStation Software A.02.02. A0: Electronic ladder; A1: bacterial RNA from the mid-exponential phase in broth culture (control); B1: bacterial RNA from infected larvae after 3-hour infection. (c) ImageJ quantification of bacterial RNA from the infected larvae (B1). Ratios of 23S:16S were calculated by the Area Under the Curve to assess the RNA integrity.

Obtaining high-quality RNAs is important in experiments for gene expression studies, such as quantitative reverse-transcription PCR (RT-qPCR), and RNA sequencing using next-generation sequencing techniques. Fig 4A shows the steps of bacterial cell harvesting from the infected larvae. Hemolymph was pooled from 40 infected larvae in order to get sufficient amounts of bacterial RNA. We noted that the host cell pellets were invisible without mixing them with the stop mix solution prior to the centrifugations. Therefore, the addition of an ice-cold stop mix solution facilitates the precipitation of host cells, as well as protects the RNA from degradation. Cell-free hemolymph can be obtained via multiple centrifugations at low speeds to remove the host cells. However, we noticed a thin layer of host cells remaining at the top layer of the supernatant (Fig 4A). Repeated centrifugations can significantly reduce these host cells at low speeds. We recommend using Tri-RNA to isolate the RNA by phenol-chloroform phase separation and purify the RNA using a column-based extraction kit to increase the RNA yield.

The ratio of absorbance at 260 nm and 280 nm (A260/280) is commonly used as the indicator of DNA/RNA purity, where protein contamination would result in a low value of A260/280. A ratio of 1.8–2.0 indicates ‘pure’ RNA. The bacterial RNA samples isolated from the infected larvae (Lane B1) showed high A260/280 ratios of 2.093 (S1 Table), suggesting pure RNA was obtained. From Fig 4B, we observed intact bands of the bacterial 23S and 16S RNAs isolated from the infected larvae (Lane B1), in which 23S and 16S RNA bands correspond to the control (bacterial RNA isolated from broth culture). Minimal host RNA contamination was observed in the *in vivo* RNA sample (B1) due to the low amount of host cells that could not be removed completely. RNA integrity numbers (RIN) were calculated by the TapeStation software. The bacterial RNA from the infected larvae had a RIN number of 5.7, which is lower than the control (RIN number of 6.7). This could be due to the multiple bands of RNAs (host and bacterial RNAs) that were all included in the RIN calculations by the TapeStation software. To further assess the RNA integrity of the *in vivo* RNA sample, the 23S:16S ratio was manually calculated by the Area Under the Curve using ImageJ [17]. Generally, a 23S:16S rRNA ratio of approximately 1.5 indicates an intact, non-degraded RNA [18]. The *in vivo* RNA sample (B1) showed relatively high 23S:16S rRNA ratios of 1.147, compared to the control which is 1.592 (Fig 4C).

In summary, this protocol provides researchers with details on the rearing and maintenance of *G*. *mellonella* without specialised training. In addition, the protocol provides detailed procedures for performing the *G*. *mellonella* infection assays, which can be applied to assess *A*. *baumannii* virulence, as well as other bacterial species, with modifications required. Furthermore, this protocol also provides the method of bacterial RNA isolation from infected larvae for gene expression studies.

## Supporting information

S1 Table Quality assessment of bacterial RNA isolated from *G*. *mellonella* larvae infected by *A*. *baumannii* strain C98. (DOCX)

S1 File Step-by-step protocol, also available on protocols.io. (dx.doi.org/10.17504/protocols.io.n92ldpr38l5b/v1). (PDF)
