# Supplementary material for: Use of the waxworm Galleria mellonella larvae as an infection model to study Acinetobacter baumannii
Source: PLoS One. 2023 Apr 5;18(4):e0283960. doi: 10.1371/journal.pone.0283960 (PMC10075412; doi:10.1371/journal.pone.0283960)
Supplement: S1 Table — (DOCX) [file pone.0283960.s001.docx]

**Use of the waxworm *Galleria mellonella* larvae as an infection model to study *Acinetobacter baumannii***

Kah Ern Ten^1^, Nazmul Hasan Muzahid^1^, Sadequr Rahman^1,2^, Hock Siew Tan^1, 2*^

^1^ School of Science, Monash University Malaysia, Bandar Sunway, Selangor Darul Ehsan, Malaysia

^2^ Tropical Medicine and Biology Multidisciplinary Platform, Bandar Sunway, Selangor Darul Ehsan, Malaysia

* Corresponding author

Email: [tan.hocksiew@monash.edu](mailto:tan.hocksiew@monash.edu) (HST)

S1 Table. Quality assessment of bacterial RNA isolated from *G. mellonella* larvae infected by *A. baumannii* strain C98.

| Samples | A260/230 | A260/280 | Concentration (ng/μL) |
| --- | --- | --- | --- |
| Broth culture control RNA | 2.780 | 2.055 | 144.9 |
| In vivo bacterial RNA (from infected larvae) | 2.618 | 2.093 | 743.1 |
