# Supplementary material for: Use of the waxworm Galleria mellonella larvae as an infection model to study Acinetobacter baumannii
Source: PLoS One. 2023 Apr 5;18(4):e0283960. doi: 10.1371/journal.pone.0283960 (PMC10075412; doi:10.1371/journal.pone.0283960)
Supplement: S1 File — (PDF) [file pone.0283960.s002.pdf]

protocols.io

Use of the waxworm Galleria mellonella larvae as an infection model to study Acinetobacter baumannii

COMMENTS 0

RESERVED DOI:
10.17504/protocols.io.n92ldpr38l5b/v1

WORKS FOR ME 1

Kah Ern Ten[1],
Nazmul Hasan Muzahid[1],
Sadequr Rahman[1,2], tan.hocksiew[1,2]

[1]School of Science, Monash University Malaysia, 47500 Bandar Sunway, Selangor Darul Ehsan, Malaysia;
[2]Tropical Medicine and Biology Multidisciplinary Platform, 47500 Bandar Sunway, Selangor Darul Ehsan, Malaysia

Kah Ern Ten

ABSTRACT

Galleria mellonella larvae have been increasingly used in various scientific research, including microbial infection studies. They act as suitable preliminary infection models to study host-pathogen interactions due to their advantages, such as the ability to survive at 37 °C mimicking human body temperature, their immune system shares similarities with mammalians, *etc*. Here, we presented a protocol for simple rearing and maintenance of *Galleria mellonella* in science research laboratories without requiring special instruments and specialised training. This allows the continuous supply of healthy *Galleria mellonella* for research purposes. Besides, this protocol also provides detailed procedures on the (i) *Galleria mellonella* infection assays (killing assay and bacterial burden assay) for virulence studies and (ii) bacterial cell harvesting from infected larvae and RNA extraction for bacterial gene expression studies during infection. Our protocol could not only be used in the studies of *A. baumannii* virulence but can also be modified according to different bacterial strains.

ATTACHMENTS

589-1236.docx

PROTOCOL INFO

Kah Ern Ten, Nazmul Hasan Muzahid, Sadequr Rahman, tan.hocksiew . Use of the waxworm Galleria mellonella larvae as an infection model to study Acinetobacter baumannii. **protocols.io**
https://protocols.io/view/use-of-the-waxworm-galleria-mellonella-larvae-as-a-cj97ur9n

KEYWORDS

Galleria mellonella, Acinetobacter baumannii, infection, killing assay, bacterial burden assay, RNA extraction

CREATED

Dec 09, 2022

LAST MODIFIED

Dec 21, 2022

OWNERSHIP HISTORY

Dec 09, 2022 renuka.s

Dec 20, 2022 Kah Ern Ten

PROTOCOL INTEGER ID

73759

GUIDELINES

Guidelines:

- Unhealthy *Galleria mellonella* larvae (with black spots/melanisation) should not be used for any experiments.
- Extra precautions must be taken when uncapping needles. A puncture-resistant glove is recommended.
- Always use RNase-free tubes when preparing reactions for RNA works. An RNase decontamination solution is highly recommended for surface decontamination of working areas to destroy RNases.
- RNA should always be kept on ice to avoid degradation.

References:

1. Andrea A, Krogfelt KA, Jenssen H. Methods and challenges of using the greater wax moth (*Galleria mellonella*) as a model organism in antimicrobial compound discovery. Microorganisms. 2019;7(3):85.
2. Fredericks L, Lee M, Roslund C, Crabtree A, Allen P, Rowley P. The design and implementation of restraint devices for the injection of pathogenic microorganisms into *Galleria mellonella*. PLOS ONE. 2020;15:e0230767.
3. Harding CR, Schroeder GN, Collins JW, Frankel G. Use of *Galleria mellonella* as a model organism to study *Legionella pneumophila* infection. J Vis Exp. 2013(81):e50964-e.

MATERIALS TEXT

Equipment and Consumables

- *Galleria mellonella* housing equipment:

A
1.2 L glass jars (tall and wide neck, 10 cm opening width, 18 cm
hard plastic jars with wide neck
heat mat
cloth-type voile
15 cm filter paper (NICE®)
plastic storage boxes

- Micropipettes (single channel) and micropipette tips
- 90mm Petri dishes
- 90mm Whatman® qualitative filter paper, grade 1
- Hamilton® Microliter™ 700 series syringe, model 725LT with Luer tip, capacity 250 µL
  250 µL Model 725 LT SYR Needles **Hamilton Company Catalog #80701**
- Repeating Dispenser **Hamilton Company Catalog #PB600-1**
- Sterilised Agani™ needle, 27G x 1/2" (0.40 × 13mm) (TERUMO)
- Spectrophotometer (Prim, US)
- BioDrop spectrophotometer
- Agilent TapeStation 2200
- Bio-Rad agarose gel electrophoresis equipment
- RNase-free microcentrifuge tubes (1.5 mL and 2 mL)
- Blunt-end forceps (Stainless steel and plastic materials)
- Cotton swab

Solutions/Reagents

- 10X Phosphate Buffered Saline (PBS), pH 7.4
  10X Phosphate Buffered Saline (PBS) **1st BASE Catalog #BUF-2040-10X1L**
- Luria Bertani Broth Miller (Miller Luria Bertani Broth) **Himedia Catalog #M1245**
- Luria Bertani Agar Miller (Miller Luria Bertani Agar) **Himedia Catalog #M1151**
- Leeds Acinetobacter Agar Base **Himedia Catalog #M1839**
- Digitonin **Sigma Aldrich Catalog #D141**
- N-Phenylthiourea **Sigma Aldrich Catalog #P7629**
- FavorPrep™ Tri-RNA Reagent **Favorgen Biotech Corp. Catalog #FATRR 001**
- Monarch Total RNA Miniprep Kit **New England Biolabs Catalog #T2010S**
- Agarose molecular biology grade **Vivantis Technologies Catalog #PC0701**
- 10X Tris-Borate-EDTA (TBE) Buffer pH 8.3 **1st BASE Catalog #BUF-3013**
- ViSafe Red Gel Stain (10000X in water) **Vivantis Technologies Catalog #SD0103**
- Ethanol Absolute (200 Proof) Molecular Biology Grade Fisher BioReagents™ **Fisher Scientific Catalog #BP2818100**
- Chloroform - AR [P] **Chemiz Catalog #38364**
- RNase Quiet **Nacalai Tesque Catalog #09147-14**
- Household bleach

- Milli-Q® water

SAFETY WARNINGS

Handle Tri-RNA, chloroform and digitonin with care. Consult SDS before use.

ATTACHMENTS

589-1236.docx

Galleria mellonella rearing and maintenance

1 Research-grade *Galleria mellonella* larvae were ordered in bulk from Carolina Biological (US).

2 Set up *Galleria mellonella* housing according to Figure 2.

Note

The glass jars must be cleaned (e.g., dishwasher detergent) and autoclaved after use.

3 Fill 2/3 of the larvae jar with the freshly prepared medium.

3.1 Ingredients of artificial diet (per jar):

A	B
NESTLE CERELAC® Infant Cereals Multi Grain & Ga	83.3 g
pure honey	20 g
99.8% glycerol	20 g
instant baker yeast	2.3 g

3.2 Mix well in a clean plastic container using a spatula.

Note

Prepared food can be stored at 4 °C for not more than 3 days.

4 Transfer healthy larvae from the container provided by the vendor individually into a new glass jar with fresh medium, and cover with a layer of cloth-type voile.

Note

We recommend using a needle to make holes in the lid as larvae could escape from large holes. This can be done by burning the needle using fire and drilling the lid with the desired size of holes.

5 Place the glass jars above a heating mat with temperature controlled at 32 °C ± 2 °C with humidity 44%-54% and keep them in a plastic storage box in a dark environment.

6 Add the fresh medium every 3 days and remove the sick/dead larvae from the jars to prevent the spread of diseases.

Note

Condensation should be wiped off using tissue paper to avoid fungal growth.

6.1 Dead/sick worms should be placed in a Petri dish or plastic bag and frozen at -20 °C Overnight . Discard as biological waste.

6.2 Larvae should be transferred individually to a new fresh medium when the old medium is dirty.

7 Allow larvae to grow into the last instar stage (approximately 300 mg, 3 cm long). At this stage, no food is needed.

7.1 Transfer healthy, creamy-white larvae at the 6th instar stage to a new glass jar with fresh food and kept at Room temperature in a dark environment for experimental use.

> Note
>
> The larvae must be used within 2 weeks.

8 Transfer 50 pupae (brownish colour) or larvae in the pre-pupal stage (with thick cocoons) into a moth jar using blunt-end forceps and cover with filter paper and a perforated lid.

> Note
>
> **NOTE**:
> 1. Pupae are very fragile. Therefore, extreme care is needed to avoid punctures.
> 2. Male and female moths will mate 2-3 days after they appear.

9 Female moths will lay eggs around the filter paper.

9.1 Replace the filter paper (that has eggs on it) with a new filter paper. This should be performed every 3 days to avoid the escape of newly hatched larvae.

9.2 The moth jars should be cleaned after 2 weeks the first moth appeared to avoid the escape of newly hatched larvae. This can be done by placing the moth jars in a cold room (4 °C) Overnight . Transfer the moths into a plastic bag and freeze them at -20 °C Overnight . Discard as biological waste.

10 Cut the collected filter paper (with eggs) into smaller pieces and transfer it into the egg jar with food.

- Discard areas with contamination.
- Cover the egg jar with a perforated lid.

Note

NOTE:
1. Extreme care is needed as the eggs can easily burst.
2. Applying a layer of Vaseline® petroleum jelly at the wall of the egg jars is highly recommended to prevent the newly hatched larvae from escaping.

11 Egg jars should be monitored every 3 days to ensure a continuous food supply until they grow into the adult stage.

11.1 Separate the medium into half using blunt-end forceps and put it into two jars when it is too crowded. Top up the medium with freshly prepared food.

11.2 Transferring the larvae individually into a new jar with freshly prepared food might be necessary when the medium has fungal contamination or an unusual smell.

11.3 When the larvae grow bigger, transfer medium and large larvae (approx. 1.5 cm and 2 cm) to a new jar with food and cover with cloth-type voile and perforated lids.

Galleria mellonella infection assays: Sample preparation

12 Incubate 10 randomly chosen healthy 6th instar stage larvae (200-300 mg) with creamy-white appearance and no melanisation at 37 °C, without food, in a standard bacterial incubator for one day before the experiment.

12.1 Pre-incubation at 37 °C allows the selection of more suitable larvae, where unhealthy larvae will show melanisation and/or death after the pre-incubation and will be excluded from the experiment.

13 Prepare bacterial overnight culture by inoculating 1 colony of *Acinetobacter baumannii* in 5 mL of Luria Bertani broth and incubate with shaking at 200 rpm, 37°C for 16-18 hours.

14 Cut pipette tips can be prepared by following Fredericks, Lee (2), which will be used as larvae restraint devices.

> Note
>
> The cut pipette tips can be reused. However, different sets of restraint devices should be prepared for different experimental groups.

14.1 Sterilise the cut pipette tips by immersing them in 70% ethanol Overnight , then discarding the ethanol and autoclaving.

Galleria mellonella infection assays: Killing assay

7m

15 Pellet 1 mL of the overnight bacterial culture by centrifuging at 8300 rpm, Room temperature, 00:07:00 .

16 Resuspend the bacterial pellet with 1 mL of sterile 1X phosphate buffer saline (PBS) (7.4).

7m

17 Repeat centrifugation (8300 rpm, Room temperature, 00:07:00) to pellet the bacterial culture.

18 Resuspend the bacterial pellet with 1 mL of sterile 1X PBS (7.4).

19 Measure the optical density of the bacterial culture and adjust it to the appropriate OD_{600} nm.

Note

1. Use 1X sterile PBS as the blank.
2. In this experiment, an OD_{600} nm of ~1, which is equal to 10^9 colony-forming units per millilitre (CFU/mL), was used. Inoculum is always confirmed via plating.
3. A 10-fold serial dilution of the bacterial culture might be required to determine the lethal and infection dose.

20 Wash the Hamilton syringe (model 725LT).

20.1 Wash the Hamilton syringe (model 725LT) with diluted bleach.

20.2 Then, wash it with distilled water to remove the bleach. (1/2)

20.3 Wash it with distilled water to remove the bleach. (2/2)

20.4 Attach the needle (27G) to the syringe and attach the Hamilton syringe to the Hamilton repeating dispenser (PB600-1).

Note

Hamilton repeating dispenser is recommended when the sample size is large to speed up the injection process.

21 Sterilise the larval prolegs with 70% ethanol using a cotton swab.

Note

This should be performed before the injection.

22 Place the larval tail into the wider part of the cut tip, then insert the narrow part of the cut tip to trap the larvae.

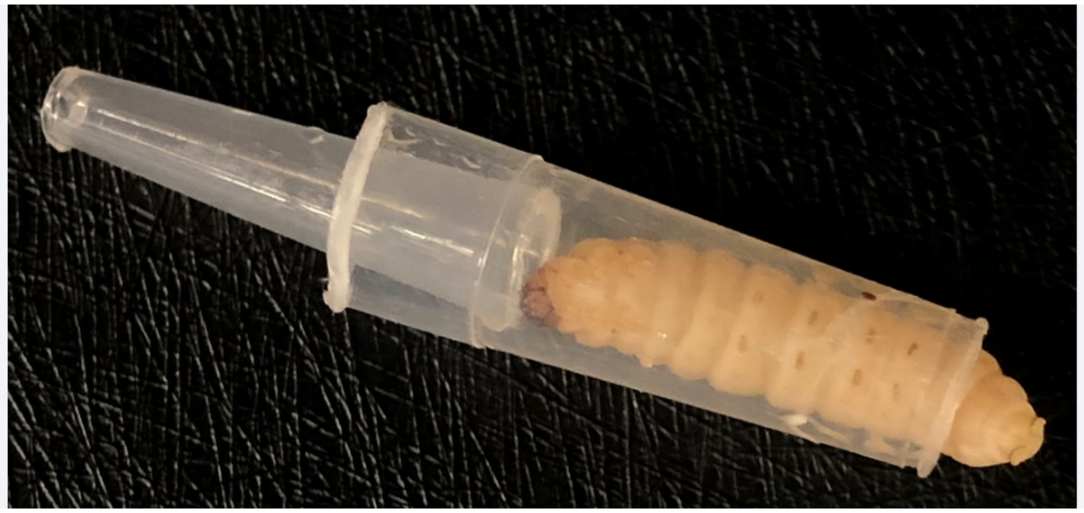

23 Inject 10 µL of bacterial suspensions with desired cell density into the last left proleg of larvae.

23.1 The needle should be visible through the larval cuticle after inserting it into the proleg.

23.2 The Hamilton syringe should be cleaned after each experimental group (step 20) to avoid being carried over to the next experimental group.

> Note
>
> Each experimental group should have separate sets of bleach and distilled water for cleaning the Hamilton syringe.

23.3 Two control groups should be used:

1. larvae injected with only sterile PBS (to assess physical trauma),
2. larvae without receiving any injections (non-manipulated control to assess background mortality).

24 Place the larvae in a sterile Petri dish lined with filter paper.

25 Incubate the larvae at 37 °C in a standard bacterial incubator and score for survival every 24:00:00 .

25.1 Larvae are considered dead when they are unresponsive to physical stimuli and melanised.

25.2 Remove larvae from the cocoon to check survival, and dead larvae should be removed from the plate at every time point to avoid the spread of diseases.

25.3 Stop the experiment when pupation occurs to avoid biases.

26 Repeat the experiments independently 3 times to get the data of 3 biological replicates (*n*=30).

> Note
>
> Only the experiments where all non-manipulated larvae survived throughout the experiment were included in the analysis.

27 Perform the Kaplan-Meier survival curves and statistical analysis (log-rank test) using GraphPad Prism software.

> Note
>
> A *p*-value of ≤0.05 indicates statistical significance.

Galleria mellonella infection assays: Bacterial burden assay (quantific...

28 Adjust overnight bacterial culture (washed twice with sterile 1X PBS, 7.4) to appropriate OD_{600} nm. Inoculum is always confirmed via plating.

29 Sterilise the larval prolegs with 70% ethanol using a cotton swab.

30 Trap the larvae in the restraint devices.

31 Clean the Hamilton syringe with diluted bleach and distilled water.

32 Inject 10 µL of bacterial suspension into the last left proleg of the larvae. Place the larvae in a sterile Petri dish lined with filter paper and incubate at 37 °C in the standard bacterial incubator.

32.1 Negative control group: larvae injected with sterile 1X PBS only.

33 Measure and record the weight of a sterile microcentrifuge tube (1.5 mL) before the hemolymph collection.

34 At each time point, randomly choose 3 larvae from the incubated larvae.

10m

35 Anaesthetise them On ice in a 15 mL centrifuge tube for 00:10:00 .

30s

36 Sterilise the larval surface by immersing them in 70% ethanol for 00:00:30 , followed by washing.

36.1 Washing with sterile distilled water to remove the residual ethanol. (1/2)

36.2 Washing with sterile distilled water to remove the residual ethanol. (2/2)

37 Make an incision by puncturing the cuticle between the second and third proleg using a sterile 27G Terumo needle. Squeeze the larvae with sterile plastic forceps (sterilised with diluted bleach and 70% ethanol) and collect the hemolymph immediately from the puncture site via pipetting.

> Note
>
> 1. The Terumo needle can be reused for the larvae from the same experimental group. Different experimental groups should use different needles to avoid contamination.
> 2. Healthy larvae have clear yellowish hemolymph, while infected larvae have melanised hemolymph.

38 Pool the hemolymph from 3 larvae into the weighted microcentrifuge tube.

> Note
>
> The hemolymph should always be kept On ice .

39 Incubate with 1 µL of digitonin (5 mg/mL) at Room temperature to lyse haemocytes and release intracellular bacteria (3).

> Note
>
> - This should be done under sterile conditions.
> - It is recommended to perform the experiment in a Class II Biosafety Cabinet.

40 Perform 10-fold serial dilution on the collected hemolymph and plate 100 µL of the bacteria with appropriate dilution onto Leeds Acinetobacter agar.

20h

41 Incubate the agar plate at 37 °C for 20:00:00 .

42 Count the number of bacterial colonies and calculate the CFU/larva by normalising to the weight of hemolymph extracted.

Note

Only the experiments with no colonies obtained from the PBS-injected control were used for analysis.

43 Perform the experiments in three independent replicates.

44 Plot the bacterial growth curve *in vivo* using GraphPad Prism software.

Bacterial harvesting and RNA extraction from infected larvae: Sample...

45 Inoculate a single colony of bacteria in 5 mL of Luria-Bertani broth and incubate for 16-18 hours with continuous shaking at 200 rpm, 37°C .

20h

46 Incubate 40 healthy larvae (6th instar stage, 200-300 mg) with a creamy-white appearance at 37 °C Overnight .

Note

1. It is recommended to prepare extra 5 to 10 larvae because sick larvae will die after pre-incubation.
2. The number of larvae can be adjusted according to the infection stage to get enough bacterial cells from the larvae.

Bacterial harvesting and RNA extraction from infected larvae: Bacteri...

7m

47 Spin 1 mL of the overnight bacterial culture at 8300 rpm, Room temperature, 00:07:00 .

48 Resuspend in 1 mL sterile 1X PBS.

7m

49 Spin the bacterial culture again at 8300 rpm, Room temperature, 00:07:00 , and resuspend in 1 mL sterile 1X PBS.

50 Adjust the bacterial culture to the appropriate optical density. Inoculum is always confirmed via plating.

51 Sterilise the larval prolegs with 70% ethanol using a cotton swab.

52 Inject 10 µL of bacterial suspension into the last left proleg of the larvae using a Hamilton syringe (model 725LT) with a 27G Terumo needle.

3h

52.1 Place the larvae in a sterile Petri dish lined with filter paper and incubate at 37 °C in the standard bacterial incubator for 03:00:00 .

> Note
>
> Incubation time can be determined from the bacterial burden assay (bacterial growth *in vivo*) depending on the stage of infection required for the studies.

53 During the incubation time, prepare a stop mix solution (95% absolute ethanol: 5% Tri-RNA) in a 2 mL RNase-free microcentrifuge tube and keep it at -20 °C . Pre-cool microcentrifuge to 4 °C .

> Note

1. Stop mix solution should be prepared freshly on the day of the experiment. Keep On ice .
2. The volume of stop mix solution = 0.2 volume of the total harvested hemolymph.

30s

54 At the desired time point, sterilise the larval surface by immersing the larvae in 70% ethanol for 00:00:30 , followed by rinsing two times with sterile distilled water.

55 Extract the hemolymph from the infected larvae using a Terumo 27G needle by puncturing the larval cuticle between the second and the third prolegs. Collect the hemolymph immediately from the punctured site and pool it into the microcentrifuge tube with ice-cold 0.2 volume of stop mix solution.

Note

1. The extracted hemolymph should always be kept On ice throughout the extraction process to prevent the melanisation of the hemolymph. The extraction should be performed quickly to avoid the degradation of RNA and to capture the gene expression accurately.
2. Digitonin is unnecessary if intracellular bacteria from the haemocytes are not needed for studies.

5m

56 Incubate the hemolymph-stop mix solution mixture at Room temperature for 00:05:00 .

5s

57 Gently vortex for 00:00:05 .

5m

58 Centrifuge at 2300 rpm, 4°C, 00:05:00 . Collect the supernatant and transfer it into a 1.5 mL RNase-free microcentrifuge tube.

Note

Avoid touching the host cell pellet.

59 Repeat step 58 until no or very little host pellet is obtained.

Note

This is necessary to minimise host RNA contamination.

15m

60 Pellet the bacterial cells from the supernatant by centrifugation at 10000 rpm, 4°C, 00:15:00 . Discard the supernatant.

5s

61 Immediately resuspend the bacterial cell pellet in 1 mL of tri-RNA. Homogenise the sample by gentle vortex for 00:00:05 or via repetitive pipetting.

62 Store at -80 °C or proceed to RNA extraction.

49m 30s

Bacterial harvesting and RNA extraction from infected larvae: Bacteri...

63 Thaw the sample On ice .

Note

This usually takes about 5-10 minutes, depending on the sample volume.

63.1 Pre-cool the microcentrifuge to 4 °C .

5m

64 Incubate at Room temperature for 00:05:00 .

65 Add 200 µL of chloroform into the mixture. Mix by inversion until a milky pink mixture is obtained.

66 Incubate at Room temperature for 00:05:00 . 5m

67 Centrifuge at 13000 rpm, 4°C, 00:15:00 . 15m

68 Collect the aqueous transparent supernatant into a 2 mL RNase-free microcentrifuge tube.

Note

Avoid touching the middle layer.

69 Add an equal volume of 95% ethanol (molecular grade).

70 Extract RNA according to the manufacturer's manual (Monarch® Total RNA Miniprep Kit, NEB) as described below:

70.1 Load the mixture onto the RNA purification column.

70.2 Centrifugation at 16000 x g, 00:00:30 . Discard the flowthrough. 30s

70.3 On-column DNase I treatment: 15m 30s

- Add 500 µL RNA Wash Buffer and centrifuge for 00:00:30 . Discard flow-through.
- Add DNase I mixture (5 µL DNase I with 75 µL DNase I Reaction Buffer) onto the column.

- Incubate for 00:15:00 at Room temperature .

70.4 Add 500 µL RNA Priming Buffer and centrifugate at 16000 x g, 00:00:30 . Discard flow-through. 30s

70.5 Add 500 µL RNA Wash Buffer and centrifugate at 16000 x g, 00:00:30 . Discard flow-through. 30s

70.6 Add 500 µL RNA Wash Buffer and centrifugate at 16000 x g, 00:02:00 . 2m

70.7 Transfer the column to a 1.5 mL RNase-free microcentrifuge tube.

70.8 Load 20 µL of RNase-free water onto the column and incubate for 00:05:00 at Room temperature . 5m

70.9 Centrifugation at 16000 x g, 00:00:30 to elute the RNA. 30s

71 Assess the RNA quality by gel electrophoresis (1% agarose gel) and Agilent TapeStation 2200 and measure the absorbance values (A260/230 and A260/280) and concentration using a BioDrop spectrophotometer.

72 Store the RNA at -80 °C or place it On ice for immediate downstream applications.
